# Alzheimer’s disease cerebrospinal fluid biomarkers are not influenced by gravity drip or aspiration extraction methodology

**DOI:** 10.1186/s13195-015-0157-7

**Published:** 2015-11-19

**Authors:** Alan Rembach, Lisbeth A. Evered, Qiao-Xin Li, Tabitha Nash, Lesley Vidaurre, Christopher J. Fowler, Kelly K. Pertile, Rebecca L. Rumble, Brett O. Trounson, Sarah Maher, Francis Mooney, Maree Farrow, Kevin Taddei, Stephanie Rainey-Smith, Simon M. Laws, S. Lance Macaulay, William Wilson, David G. Darby, Ralph N. Martins, David Ames, Steven Collins, Brendan Silbert, Colin L. Masters, James D. Doecke, the AIBL Research Group

**Affiliations:** The Florey Institute of Neuroscience and Mental Health, The University of Melbourne, Victoria, 3010 Australia; Centre for Anaesthesia and Cognitive Function, Department of Anaesthesia and Perioperative Pain Medicine, St Vincent’s Hospital, Melbourne, Australia; Alzheimer’s Australia Victoria, 155 Oak Street, Parkville, Victoria 3052 Australia; Centre of Excellence for Alzheimer’s Disease Research & Care, School of Medical Sciences, Edith Cowan University, Joondalup, Western Australia Australia; Sir James McCusker Alzheimer’s Disease Research Unit (Hollywood Private Hospital), Perth, Western Australia Australia; Department of Pathology, University of Melbourne, Parkville, 3010 Australia; CSIRO Preventative Health Flagship, Parkville, Victoria 3010 Australia; CSIRO Computational Informatics/Australian e-Health Research Centre, Brisbane, Queensland 4029 Australia; National Ageing Research Institute, Parkville, Victoria 3050 Australia

## Abstract

**Introduction:**

Cerebrospinal fluid (CSF) biomarkers, although of established utility in the diagnostic evaluation of Alzheimer’s disease (AD), are known to be sensitive to variation based on pre-analytical sample processing. We assessed whether gravity droplet collection versus syringe aspiration was another factor influencing CSF biomarker analyte concentrations and reproducibility.

**Methods:**

Standardized lumbar puncture using small calibre atraumatic spinal needles and CSF collection using gravity fed collection followed by syringe aspirated extraction was performed in a sample of elderly individuals participating in a large long-term observational research trial. Analyte assay concentrations were compared.

**Results:**

For the 44 total paired samples of gravity collection and aspiration, reproducibility was high for biomarker CSF analyte assay concentrations (concordance correlation [95%CI]: beta-amyloid1-42 (Aβ42) 0.83 [0.71 - 0.90]), t-tau 0.99 [0.98 - 0.99], and phosphorylated tau (p-tau) 0.82 [95 % CI 0.71 - 0.89]) and Bonferroni corrected paired sample t-tests showed no significant differences (group means (SD): Aβ42 366.5 (86.8) vs 354.3 (82.6), *p* = 0.10; t-tau 83.9 (46.6) vs 84.7 (47.4) *p* = 0.49; p-tau 43.5 (22.8) vs 40.0 (17.7), *p* = 0.05). The mean duration of collection was 10.9 minutes for gravity collection and <1 minute for aspiration.

**Conclusions:**

Our results demonstrate that aspiration of CSF is comparable to gravity droplet collection for AD biomarker analyses but could considerably accelerate throughput and improve the procedural tolerability for assessment of CSF biomarkers.

## Introduction

In 2011, proceedings from the National Institute on Aging - Alzheimer’s Association workgroups on diagnostic guidelines for Alzheimer’s disease (AD) recommended the addition of biomarkers as an important component of diagnosis [1]. Neuroimaging and cerebrospinal fluid (CSF) sampling are able to identify individuals with high likelihood of AD pathology. Amyloid-PET imaging can detect neocortical accumulation of fibrillary amyloid up to 30 years prior to first symptoms of AD [2] and CSF biomarker assays demonstrate abnormalities at least 20 years prior to the expected age of onset in dominantly inherited AD pedigrees [3].

The CSF in established AD is characterized by a decrease in Aβ42 and increases in total tau (t-tau) and tau phosphorylated at threonine 181 (p-tau). The diagnostic performance of these CSF biomarkers to discriminate AD from non-demented older individuals is high, with sensitivity and specificity figures of 80 %–90 % [4]. In addition to the high sensitivity and specificity, CSF analysis has several advantages as a biomarker of choice. Lumbar puncture is easy to perform, does not require expensive and sophisticated equipment and does not require the use of expensive radio-ligands. Furthermore, it provides a simple fluid matrix that allows AD biomarker interrogation and potential additional evaluation of other analytes, as well as routine biochemistry and microscopy. Herskovits and Growden have called for the widespread establishment of lumbar puncture clinics to enable the routine use of CSF both for diagnosis of AD and the monitoring of the CSF response to therapeutic trials [5]. Adverse events with lumbar puncture (LP) are low with technical optimization [6]. No major complications from LPs conducted in memory clinics were reported in 1,089 patients, and mild post-LP headaches were reported in only 28 (2.6 %) of the patients [7].

Considerable variability in absolute concentrations of AD biomarkers has been reported by different centers using the same assay, leading to different cutoff values [8]. A quality control program has been established to decrease variability across centers [9]. An important adjunct to this has been the recommendation of systematic pre-analytical handling by the Alzheimer’s Biomarkers Standardization Initiative (ABSI) [10]. This consensus statement outlined how pre-analytical management of CSF should be undertaken in order to standardize all aspects of sample handling until the point of analysis. Most of the recommendations were based on expert opinion rather than evidence. A commitment was made to provide evidence for unanswered questions such as the effect of tube composition on assay results.

A key issue which was not considered in the ABSI statement was the methodology for CSF collection. During LP CSF can be collected by allowing it to drip into the collecting tube (gravity drip) or by aspiration with a syringe (syringe aspiration). Proponents of gravity drip maintain that when a syringe is used to aspirate CSF, the extra surface area of the syringe (even when it is polypropylene) may adsorb analytes and thus influence assay results, while others believe that if a suitable polypropylene syringe is used, the resulting assays for the biomarkers of AD will not be affected. Consequently, some investigators use gravity flow in the belief that this will minimize this effect [11]. Gravity can also be used to fill the manometer tubing for measuring CSF pressure and then emptying the fluid in the tubing into polypropylene containers [7] (personal communication Kaj Blennow). Other investigators routinely use syringe aspiration. The method of CSF collection is an important issue because taking volumes of CSF greater than 10 mL by gravity drip is time consuming and can be uncomfortable for the participant. The time taken could be an impediment to routine CSF sampling if higher throughput is desired for both diagnosis and monitoring of AD.

To investigate potential effects of collection methodology we compared gravity drip collection (directly from the needle) with negative pressure aspiration using a polypropylene syringe when assaying AD CSF biomarker concentrations, during the same lumbar puncture for each subject. All other pre-analytical and analytical aspects of the procedures were identical. We hypothesized that analyte concentrations would be in agreement between techniques.

## Methods

### Participants

A total of 54 participants (38 healthy controls (HC), eight with mild cognitive impairment (MCI) and eight with AD) from the Australian Imaging Biomarker and Lifestyle (AIBL) study of aging were evaluated using standardized LP. Description of the psychometric test battery and characterization of AIBL participants at baseline has been reported previously [12]. All procedures were carried out with institutional human ethics approval (St Vincent’s Hospital, Fitzroy, Victoria, 3065, Australia) and participant/carer informed consent.

### Lumbar puncture

CSF was collected by LP in the morning from overnight fasted participants using protocols aligned with the ABSI [10]. All LPs were performed using a strictly aseptic technique (gloves, gowns, masks and sterile draping) with the subjects in the sitting position. The LP was attempted using a Temena (Polymedic®, EU, temena.com) spinal needle micro-tip (22/27G x 103 mm) (CAT 21922–27). If there was difficulty using this fine needle, a RapID set pencil point spinal needle, 25G (Smiths Medical ASD, Inc, Keene, NH, USA) was used. The procedure was constrained by the AIBL CSF collection protocol which required gravity drip collection, so all aspiration samples for this study were taken after gravity drip collection. Up to 6 mLs of CSF was initially aspirated for routine microbiological and biochemical assessment, as well as concurrent studies, after which 8 mLs of CSF was collected by gravity flow into a 15 mL polypropylene tube (Greneir Bio-One188271), and placed immediately onto wet ice. At the completion of the gravity collection, a polypropylene syringe (BD, North Ryde, NSW, Australia; 2 ml syringe, ref 302204) was then used to aspirate 2 mL of CSF, which was then transferred to a polypropylene tube on ice. Samples were processed within one hour and kept at 4 °C during transport to the laboratory. The CSF was centrifuged (2,000 x g, 4 °C, for ten minutes) and the supernatant transferred to a new polypropylene tube and gently inverted. The CSF was then aliquoted in 300 μL volumes into Nunc cryobank polypropylene tubes (NUN374088). Samples were transferred to dry ice immediately, and then into liquid nitrogen vapor tanks within one hour and only thawed once immediately before analysis. All participants were contacted by telephone the following day, and also provided a phone number if there were any concerns such as headache or backache.

### xMAP biomarker assay

All samples were measured in duplicate using the AlzBio3 xMAP assay (Innogenetics, N.V. Ghent, Belgium) according to the included protocol for human CSF Aβ42, t-tau and p-tau. A total of four assay kits were used to collect the data spread over several days, and all steps were conducted by the same operator blinded to participant and CSF collection method, using multichannel pipettes and a manual wash/vacuum manifold. The same kit batch-number was used for all assay plates used in the study and the participant samples were distributed randomly on the analysis plates. Briefly, all reagents, standards, controls and samples were brought to room temperature and vortex-mixed immediately before the assay. Coated beads were vortexed for three minutes in a sonicating water bath. All working solutions were diluted in Milli-Q H_2_O or supplied diluents according to the kit instructions. The filter plate was washed once using 225 μL/well of 1x wash buffer and vacuum aspirated immediately before use. A total of 100 μL of bead suspension (3,000 beads/analyte) was added to each well and the plate vacuum aspirated. Then 25 μL of the conjugate working solution was added to each well. A total of 75 μL of standards, kit and pooled CSF controls and samples were added to the plate in duplicate. A buffer blank was also included. The filter plate was sealed, the bottom of the plate was dabbed dry and the plate was wrapped in aluminum foil and incubated on an orbital plate shaker at room temperature (21–23 °C). The plate was incubated overnight (at least 14 hours). On the second day, the remaining reagents were prepared at room temperature. The filter plate was aspirated and then washed with 225 μL of 1x wash buffer, three times. A total of 100 μL of diluted detection reagent was then added to each well and the plate re-covered in foil for one hour on an orbital plate shaker. The filter plate was aspirated and washed with 225 μL of 1x wash buffer, three times. Then, 100 μL of read solution was added to each well and the plate was finally incubated on an orbital plate shaker for five minutes covered in foil, at room temperature. The sample concentrations were then quantified using a Bio-Rad Bioplex 200 instrument (Bio-Rad Laboratories, USA) using 5PL logistic regression. The data were fitted to calibration curves constructed with the median fluorescence values for each replicate of the standards. Concentrations were determined by sigmoidal curve fitting. Data from duplicate sample measurements for each individual’s CSF sample that had a percentage coefficient of variance (%CV) above 20 % (as recommended by the manufacturer) were discarded and the samples were reanalyzed. For all plates, the internal standard control duplicate analyses were within the accepted %CV.

### Statistical analysis

Biomarker agreement was assessed using the Concordance Correlation test, with a correlation coefficient close to 1 representing perfect repeatability. Bland-Altman plot analysis was conducted to assess agreement between methods [13]. To determine whether there were any significant differences in CSF analyte levels between collection methods, the data were analyzed using a paired sample t-test. A Bonferroni adjustment for multiple comparisons was used for *p*-value assessment, with test results compared against 0.0167 (0.05/3). Statistical analyses for concordance and sample mean differences were performed on the original set of 35 samples where the %CV was less than 20 %, as well as the complete set of 44 samples to determine whether there were any comparable differences between the original assay set and the repeated samples. Linear models between CSF collection methods were compared using analysis of variance. Demographic characteristics were assessed using the independent samples t-test (age), Chi square analyses (*APOEε4* and sex), and Kruskal Wallis tests (Clinical Dementia Rating (CDR) score and Mini–Mental State Examination (MMSE) score). All statistical analyses were conducted using the R software version 3.0.2.

## Results

Of the 54 participant samples, 19 failed quality control for one or more of the multiplexed analytes, with %CV greater than 20 %. Of these 19, nine samples were re-run and performed with < 20 % %CVs and were included in the final analysis using a total of 44 participant samples. Demographic characteristics of the participants are shown in Table 1. There was no significant difference in ages between diagnostic classifications (*p* = 0.40) or in the distribution of males and females between clinical classifications (*p* = 0.41). There were more *APOEε4* carriers in the AD group; however, this was not statistically significant (*p* = 0.20). As expected, the CDR scale was significantly higher and the MMSE score significantly lower in the MCI and AD groups (*p* < 0.0001). The mean inter-assay %CV based on the lowest concentration included standards was 11.6 % for Aβ42, 7.26 % t-tau, and 11.29 % for p-tau.

**Table 1:** Sub-cohort demographics

	HC	MCI	AD	*p* - value
N	31	6	7	
Age (years)	71.8 (5.6)	68.3 (4.5)	71.3 (7.1)	0.40
Sex (F%)	58 %	50 %	28 %	0.41
*APOEε4* %	19 %	0 %	43 %	0.20
MMSE (median IQR)	29 (1.5)	26 (2)	22 (3)	<0.0001
CDR (median IQR)	0 (0)	0.5 (0)	1 (0.25)	<0.0001

*p*–value determined by t-test (age), Chi square analyses (*APOEε4* and sex) and Kruskal-Wallis tests (CDR score and MMSE)

*N* number, *HC* health control, *MCI* mild cognitive impairment, *AD* Alzheimer’s disease, *APOEε4* apolipoprotein epsilon 4 allele, *MMSE* Mini–Mental State Examination, *CDR* Clinical Dementia Rating, *IQR* inter-quartile range

**Fig. 1:**
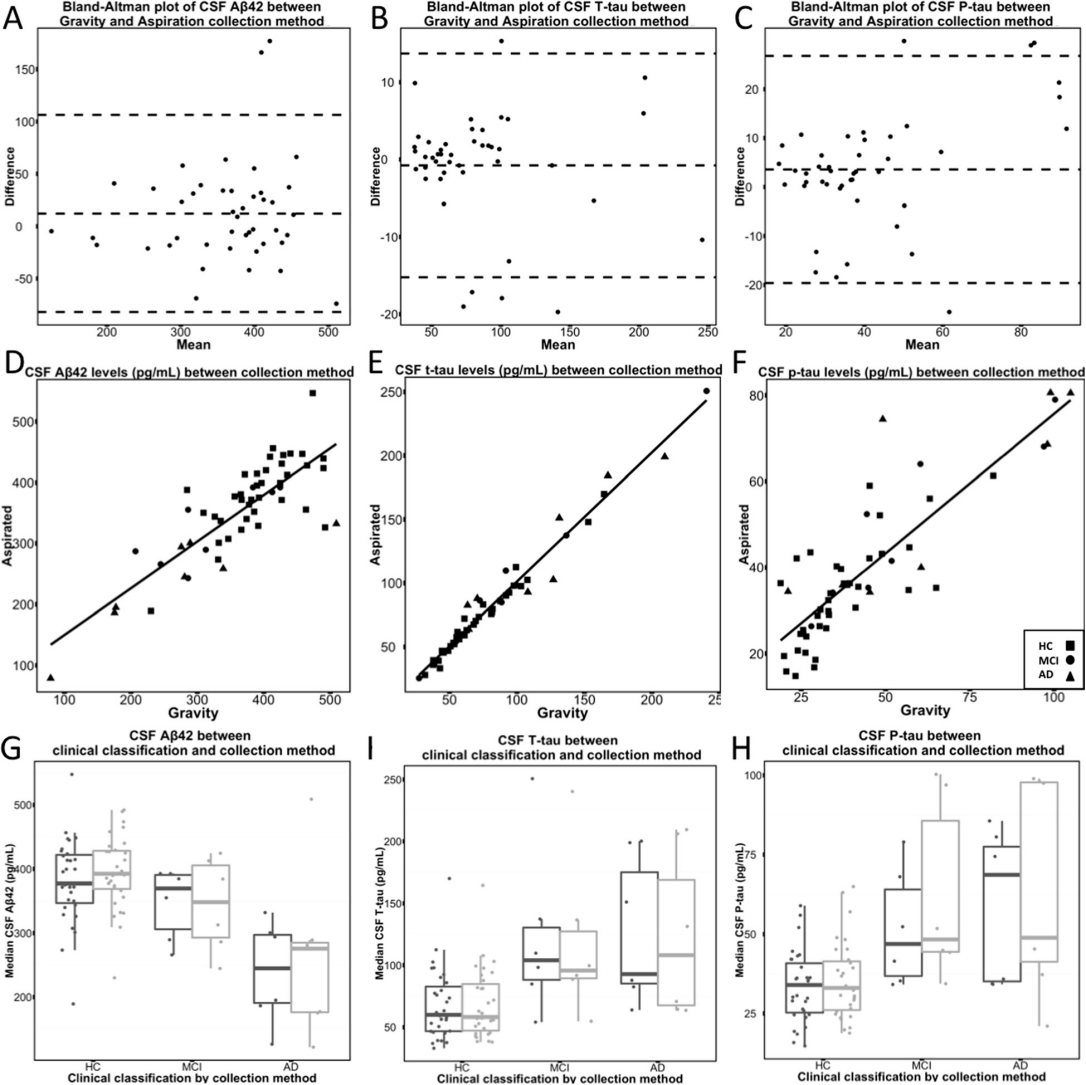
**a**-**c** Bland-Altman plots confirming good agreement between the two collection methods. **d**-**f** Concordance correlations between the methodologies showed strong reproducibility for Aβ42, t-tau, and p-tau. **g**-**i** The median and interquartile range for each biomarker value divided by each clinical classification (gravity = black; aspiration = grey)

Using the complete sample (n = 44), concordance correlations between CSF collection methods showed strong reproducibility for Aβ42, t-tau, and p-tau (0.83 [95 % Confidence Interval (CI 0.71 - 0.90], 0.99 [95 % CI 0.98 - 0.99], and 0.82 [95 % CI 0.71 - 0.89], respectively). Reducing the sample to only those with a < 20 % %CV in the original assay (n = 35) performed similarly compared to that of the complete sample (Aβ42 0.87 [0.77 - 0.93], t-tau 0.99 [95 % CI 0.98 - 0.99], and p-tau 0.86 [95 % CI 0.76 - 0.93], respectively). Bland-Altman plots also displayed good agreement between the two collection methods (Fig. 1g, h, i). Table 2 shows the means (SD) for the three biomarker analyte quantifications.

**Table 2:** Mean values of AD CSF biomarkers, gravity versus aspiration

	N	Aβ42 [pg/mL]			t-tau [pg/mL]			p-tau [pg/mL]		
		*g*	*a*	*p*-value	*g*	*a*	*p*-value	*g*	*a*	*p*-value
Complete	44	366.5 (86.8)	354.3 (82.6)	0.10	83.9 (46.6)	84.7 (47.4)	0.49	43.5 (22.8)	40.0 (17.7)	0.05
HC	31	394.6 (56.4)	381.6 (65.8)		68.6 (27.9)	68.2 (28.8)	0.57	35.3 (12.1)	33.4 (11.5)	
MCI	6	344.2 (73.5)	346.8 (55.7)		118.6 (65.1)	122.5 (68.6)	0.31	62.1 (28.9)	51.7 (18.4)	
AD	7	261.38 (126.9)	240.04 (73.9)		121.92 (63.8)	125.27 (57.4)	0.58	63.86 (33.3)	59.08 (23.3)	

Mean (standard deviation)

*g* gravity fed; *a* aspiration, *t-tau* total tau, *p-tau* phosphorylated-tau

*p*-values are unadjusted from Paired Sample T-tests

For the complete cohort, we found no significant difference (post-correction for multiple comparisons) in mean Aβ42, t-tau, and p-tau levels between gravity-fed and aspiration collection methods (Aβ: gravity: 366.5 (86.8) vs aspiration: 354.3 (82.6) *p* = 0.10; t-tau: gravity: 83.9 (46.6) vs aspiration: 84.7 (47.4), *p* = 0.49; and p-tau gravity: 43.5 (22.8) vs aspiration: 40.0 (17.7), *p* = 0.05). Analyses of the reduced set (N = 35) found similar results for each of the analytes (Aβ: gravity: 373.4 (88.0) vs aspiration: 366.5 (84.8) *p* = 0.35; t-tau: gravity: 82.8 (48.5) vs aspiration: 82.3 (48.7), *p* = 0.64; and p-tau gravity: 41.9 (22.7) vs aspiration: 38.5 (18.6), *p* = 0.07). Assessment of intra-classification means showed only small changes in biomarker level between gravity and aspiration collection methods; t-tests were not performed due to low sample size (Table 2, Fig. 1a, b, c). Gravity drip collection versus aspirated CSF was also plotted differentiating clinical classification (Fig. 1d, e, f).

We also classified each participant using the standardized cut-off scores of our laboratory (thresholds for Aβ, t-tau, and p-tau were 281, 106, and 46, respectively, ± 5 % error) and dichotomized using the 10^th^ and 90^th^ percentiles (Li et al., personal communication). Using this method, only one participant was classified differently between collection methods (with a 6 % difference between collection methods).

In four of the 54 participants, it proved difficult to find the CSF with the fine #27 gauge needles and the 25 gauge pencil point needle was successfully employed. Of the 54 subjects, two complained of mild headache when questioned the following day by telephone follow-up. Both headaches resolved quickly with mild analgesics (one with acetaminophen, the other with ibuprofen). A further participant complained of nagging back pain, but had suffered back pain for many years and the pain following the LP was not clearly different to pre-existing pain. The duration for CSF extraction was a mean of 10 (range 5–23) minutes. A total of 16 mLs was taken from all participants except one in whom only 11 mL was extracted because of slow gravity drip (22 minutes) during which the participant became tired (see Table 3). All aspiration samples were extracted in less than one minute.

**Table 3:** Parameters of the sample collection and reported adverse incidents

Age (years)	Number	Volume collected^a^ (mLs)	Time of day at collection	Total time taken to collect the CSF (min)	Average time taken to collected via gravity (min)	Average time taken to collect via aspiration (min)	Length of Stay	Reported adverse incidents
72.49 (5.54)	N = 54	16	9-10 am	10.87 (4.12)^b^	10-15	0.5-1	4 hours post LP	2 reports of post-LP headaches lasting more than 24 hours. 4 LPs initially failed with 27 g needles and were successful when the 25 g needles were used.

Data represents the average and (standard deviation)

*CSF* cerebrospinal fluid, *LP* lumbar puncture

^a^N = 1 LP resulted in only 11 mL total volume collected via gravity and 1 mL via aspiration

^b^One subject’s total time was not recorded

## Discussion

The current study confirms there is strong agreement in CSF Aβ42, t-tau, and p-tau levels between gravity feed and aspiration collection methods. Aβ42 and p-tau had slightly lower concordance correlation coefficients (>0.8) due to increased assay variation, causing a reduced agreement statistic as compared with t-tau. Analyses of the three biomarkers via paired samples t-tests showed no significant difference between collection methods; however, there was a trend towards a difference in p-tau levels (largely driven by the AD group) prior to adjustment for multiple comparisons. We included MCI and AD controls to address the issue of varying HC levels of AD pathology potentially influencing the dynamic levels of each specific analyte or the way they behave *ex vivo*. This is the first time to our knowledge that a direct comparison of aspiration and gravity fed collection has been reported.

Importantly, all CSF samples were taken sequentially from the same LP and all analyzed simultaneously. The CSF samples were thus not subjected to any variation in pre-analytical handling other than the method of collection and, furthermore, were not exposed to technical variations, which may have contributed to previously seen variation observed between laboratories, underscoring the accuracy of the results.

The method of CSF collection is an important step in the pre-analytical handling of CSF samples. While some investigators routinely use gravity, others use aspiration. Gravity drip has the drawback of unpredictable variation of collection times and may potentially take considerably longer than aspiration, thereby reducing feasibility in busy clinics. In dementia evaluation settings, the longer duration of CSF acquisition can be a particular problem for a patient with memory impairment or dementia since repeated reassurance and explanation may be required. Our results demonstrate that syringe aspiration does not have a significant effect on analyte concentrations and, therefore, should be acceptable and allow predictable and more rapid CSF collection.

In our study, post lumbar puncture headaches using the #27 and #25 gauge needles occurred in 2/54 procedures (4 %) of patients consistent with previous reports when using small caliber needles [14]. This is markedly below that reported when #24 or #22 Sprotte needles are used (eg., 22 % [15]), but the mean age of their samples was 40.2 (10.1) years compared to the mean age of our sample of 72.5 (5.5) years. Hence, an added bonus to syringe aspiration is that negative pressure sampling allows the use of small caliber spinal needles decreasing the risk of headache. We emphasize that our results pertain to the use of small caliber spinal needles in an elderly patient population. The use of larger caliber spinal needles may lead to a higher incidence of headache, presumably due to more rapid shifts in CSF. Furthermore, whilst gravity collection of CSF can take more than 20 minutes, aspiration can collect the usual 10–20 mLs of CSF required in a few minutes.

Reasons for a preference of gravity flow collection over aspiration in some centers may be historical. A belief that aspiration may cause radicular pain by traumatizing local nerve roots [16] appears unfounded, and might be even less common with smaller caliber needles. On the other hand, syringe aspiration may be inappropriate whenever CSF pressure needs to be measured with manometer tubing which then allows collection of tube contents via gravity. Measurement of CSF opening pressure, though, is not likely to be necessary when a CSF sample is only required for the purpose of AD biomarker analysis.

To our knowledge, this is the first report of concordance of CSF biomarkers when CSF is collected either by standard gravity drip or syringe aspiration. In particular, more rapid collection by aspiration suggests that wider adoption of aspiration is feasible and may become the preferred means of CSF collection for the detection of AD CSF profiles. Shorter duration should help facilitate acceptance both by patients undergoing the procedure and also staff conducting the LP. Indeed, the implications of reducing the time of the extraction of the CSF from around ten minutes for gravity feed to approximately one minute for aspiration, may allow the procedure to be suitable for mass screening as advocated by Herskovits [5].

These results also indicate that the use of an extra polypropylene contact step (in the syringe) used in the aspiration technique made no significant difference to the CSF biomarker concentrations, despite prior concerns [17]. With the increasing use of atraumatic LP needles, which dramatically reduce the incidence of post-LP headache [18, 19], and the escalating need for accurate diagnosis of early AD, our results add support for aspiration as a time saving and benign method for routine CSF collection for AD biomarker analyses.

A limitation to the study was that all aspirations were performed subsequent to gravity drip collection. This was because the protocol for the AIBL study demanded that samples be taken by gravity drip, and, therefore, it was only after the required samples had been obtained for AIBL that we were able to take the aspiration sample. Although we consider it unlikely that reversing the CSF collection technique order will significantly influence analyte concentrations, formal assessment of this issue could be the subject of future studies.

A further limitation in this study was the relatively small sample size, with varying proportions of variation per analyte, and insufficient power to evaluate analyte concordance within clinical classifications. Further studies with larger samples sizes are required to evaluate whether clinical classification influences correlations between methods.

## Conclusions

The results of this study indicate that there is no significant difference in CSF AD biomarker concentrations for Aβ42, p-tau, and total tau between gravity collection and polypropylene syringe aspiration, yet aspiration is much quicker. We conclude that aspiration should be considered as the favored collection technique for CSF AD biomarker evaluation as this technique becomes more widely adopted throughout the world.

## Abbreviations

%CV: Percentage coefficient of variance
22/27G: Gauges of needles
ABSI: Alzheimer’s Biomarkers Standardization Initiative
AD: Alzheimer's disease
AIBL: Australian Imaging Biomarker and Lifestyle study of aging
APOE: Apolipoprotein E
Aß: beta-amyloid
CAT: Catalogue number
CDR: Clinical Dementia Rating
CI: Confidence interval
CSF: Cerebrospinal fluid
CSIRO: Commonwealth Scientific Industrial Research Organization.
DCRD: Dementia Collaborative Research Centres program
EU: European Union
g: Gravity (table 2)
HC: Healthy control
IQR: Inter-quartile range
LP: Lumbar puncture
MCI: Mild cognitive impairment
MMSE: Mini-Mental State Examination
NHMRC: National Health and Medical Research Council
PET: Positron emission tomography
p-tau: Phosphorylated tau
SD: Standard deviation
t-tau: Total tau
xMAP: Proprietary multiplexing technology
